# Metabolic networks of the *Nicotiana* genus in the spotlight: content, progress and outlook

**DOI:** 10.1093/bib/bbaa136

**Published:** 2020-07-14

**Authors:** Hartmut Foerster, James N D Battey, Nicolas Sierro, Nikolai V Ivanov, Lukas A Mueller

**Affiliations:** 1 Boyce Thompson Institute; 2 PMI R&D, Philip Morris Products S.A

**Keywords:** taxon-specific databases, manual curation, data quality, literature coverage, database development

## Abstract

Manually curated metabolic databases residing at the Sol Genomics Network comprise two taxon-specific databases for the *Solanaceae* family, i.e. SolanaCyc and the genus *Nicotiana*, i.e. NicotianaCyc as well as six species-specific databases for *Nicotiana tabacum* TN90, *N. tabacum* K326, *Nicotiana benthamiana*, *N. sylvestris*, *N. tomentosiformis* and *N. attenuata*. New pathways were created through the extraction, examination and verification of related data from the literature and the aid of external database guided by an expert-led curation process. Here we describe the curation progress that has been achieved in these databases since the first release version 1.0 in 2016, the curation flow and the curation process using the example metabolic pathway for cholesterol in plants. The current content of our databases comprises 266 pathways and 36 superpathways in SolanaCyc and 143 pathways plus 21 superpathways in NicotianaCyc, manually curated and validated specifically for the Solanaceae family and *Nicotiana* genus, respectively. The curated data have been propagated to the respective *Nicotiana*-specific databases, which resulted in the enrichment and more accurate presentation of their metabolic networks. The quality and coverage in those databases have been compared with related external databases and discussed in terms of literature support and metabolic content.

## Introduction

High-quality databases that store, manage and make large datasets readily accessible to a broad variety of biological analyses have taken a central place in bioinformatics [1, 2]. The ever-increasing flow of data from Omics, high-throughput and next-generation technologies, and the need to translate those raw data into meaningful biological information require the continuous development of databases, which are up to the task of handling those emerging new challenges [3]. The value of a biological database is defined by its curation status. Manually curated databases maintained by highly trained biocurators represent the gold standard of databases, which stands for high quality and verifiable data, supported by original research publications, easy access, compatibility with other resources and comparability of data that allow deeper insights and new discoveries [4].

A number of metabolic databases have been established addressing this growing need for repositories that host and manage and curate high-quality data published by the scientific community. Such databases provide not only an overview of the latest state in the respective research field, but also offer the tools to supplement experimental findings with bioinformatics analyses. A broad variety of such databases with a different focus and functionality exist. Human metabolic databases such as Reactome [5] and Recon 1 [6] focus on human proteins and their molecular functions [7] and genome-scale metabolic modeling [8, 9] to unravel the human metabolic network. MapMan [10] represents a framework of annotated metabolic, regulatory and signaling processes in plants, designed to overlay metabolic networks with Omics data obtained from multiple resources [11]. However, with regard to the coverage of general metabolism two databases stand out. One is the Kyoto Encyclopedia of Genes and Genomes, which projects biological processes from various organisms onto pathways consolidated in large network schemata [12]. The other one is the database system of the Pathway Tools suite [13, 14], creating Pathway/Genome Databases (PGDBs) that visualize and provide comments on reactions, compounds, enzymes, genes and regulatory aspects in pathways diagrams [15, 16]. The PGDBs provide a repertory of applications aiding functional analyses such as feeding Omics data to individual pathways or the entire metabolic map of the species [17–19], metabolic flux analysis [20], the pathway collage tool for the customized presentation of pathways and their components [21], SmartTable capability [22], and comprehensive search and querying options for exploring metabolic networks [23].

MetaCyc is the principal pathway database, which contains all the reference pathways used for predicting metabolic networks for organisms with annotated genomes [24]. Several thousands of derivative databases have been created since the inception of MetaCyc, but only very few of them are manually curated and maintained at the highest standard of curation, which corresponds to the Tier 1 specification of continually updated and intensively curated databases [15]. Among them is AraCyc, one of the first species-specific PGDBs created for plants [25, 26]. Whereas the overwhelming majority of derivative plant PGDBs is species-specific databases, a few databases represent higher taxonomic ranks. PlantCyc (http://www.plantcyc.org/) is a kingdom-level database, which contains the metabolic information from more than 350 plant species and is used, besides MetaCyc, as a supplementary reference database for the prediction of more plant-specific databases in the Plant Metabolic Network (PMN) [27]. The latest addition to manually curated taxon-specific databases is hosted at the Sol Genomics Network (SGN) (https://solgenomics.net/) [28, 29] on the SolCyc site (https://solgenomics.net/pages/solcyc/) and comprises, among others, the family-specific SolanaCyc database, containing all experimentally verified metabolic data of the *Solanaceae* family and the genus-specific NicotianaCyc database, which holds the corresponding metabolic information for the *Nicotiana* genus [30].

Metabolism is fundamentally important for understanding life processes. Numerous enzymatically catalyzed reactions convert a multitude of compounds within a defined network of primary and specialized metabolites that is similar but not identical between plant species. PMNs have evolved over time into a complexity unrivaled among any organisms [31]. Those networks are constantly refined and modified by underlying evolutionary pressures, which still elude our full understanding [32]. A large part of that complexity is due to the production of highly divergent specialized metabolites, which are not ubiquitously found in the plant kingdom, but are often restricted to particular plant families, genera or even species of certain lineages [33–36]. The estimated numbers of plant specialized metabolites range from more than 200 000 [37] up to 1 million different natural products [38]. Of the 1 million metabolites only about 1.4% (>14 000) have been characterized and can be measured. Even taking the more conservative estimate of 200 000 specialized metabolites into account still only amounts to merely 7% of identified compounds in plants [39].

One of the reasons for the huge diversity of specialized metabolites lies in the multi-specificity or even promiscuity of the catalyzing enzymes [40, 41], which has been developed over time [42] and reflects the remarkable adaptability of terrestrial plants conquering new habitats [43]. Enzymes catalyzing specialized metabolites often display broad metabolic activities resulting in an astounding array of natural products [44]. For instance, the gamma-humulene synthase of *Abies grandis* is able to synthesize 52 sesquiterpene olefins [45], and the gibberellin biosynthetic pathway produces 136 products, of which only a few have an established biological activity [46]. The effort to capture the diversity of plant specialized metabolism is mirrored in the curation efforts of various metabolic databases. The current versions of databases, which only contain curated pathways such as MetaCyc, PlantCyc, SolanaCyc and NicotianaCyc, have a share of specialized metabolite pathways of 30.3, 49.9, 50.0 and 35.7%, respectively. That percentage in relation to all other metabolic pathways makes specialized metabolites the compound category with the most curated entries in those databases.

In this publication, we describe the progress made in the manual curation of SolCyc databases (https://solgenomics.net/pages/solcyc/). We added one more *Nicotiana*-specific database for *Nicotiana attenuata* to the SolCyc database collection, which brings the count of manually supervised databases under the umbrella of SolCyc to two taxon-specific databases, i.e. SolanaCyc and NicotianaCyc, and six *Nicotiana*-specific databases for *N. tabacum* TN90, *N. tabacum* K326, *N. sylvestris*, *N. tomentosiformis*, *Nicotiana benthamiana* and *N. attenuata*. Using the cholesterol biosynthesis in plants as an example, we illustrate curation progress made on pathways and, consequently, the associated complementation of the inherent metabolic grid, which extends toward related pathways such as the biosynthesis of the steroidal glycoalkaloids solasodine and alpha-tomatine. We also discuss the curation flow and exchange of data with the reference database MetaCyc, look at pathway curation quality and enzyme coverage between in-house and external databases and compare the metabolic networks of the *Nicotiana*-specific databases. The curation effort in our databases has contributed to a more complete, enriched and accurate representation of their metabolic networks and will continue to enhance the range and pathway predictability of MetaCyc to which all pathways will ultimately be submitted.

## Material and methods

### Nicotiana genomes reannotation

The assemblies for *N. tabacum* accession TN90 (GCA_000715135.1), *N. sylvestris* (GCF_000393655.1) and *N. tomentosiformis* (GCF_000390325.2) were downloaded from National Center for Biotechnology Information (NCBI) assembly (https://www.ncbi.nlm.nih.gov/assembly) [A. Bombarely, unpublished results, 47, 48]. Previous messenger ribonucleic acid (mRNA) annotations for each of the genomes were downloaded from the SGN database (ftp://ftp.solgenomics.net/genomes/). Additionally mRNA sequences were complemented with publicly available Sanger ESTs from NCBI GenBank and assembled 454 ESTs from SGN (ftp://ftp.solgenomics.net/transcript_sequences/by_experiment/decipher_ntab/assembly/) [49]. De novo repeats were analyzed using RepeatModeler v1.0.8 (default parameters). De novo repeats and mRNA were used to re-annotate the *Nicotiana* genomes using Maker-P [50] with the default parameters. A total of 72,866, 37,162 and 36,509 gene models and 69,211, 35,553 and 34,378 protein coding genes were annotated for the *N. tabacum*, *N. sylvestris* and *N. tomentosiformis* genomes, respectively. Functional annotation was performed searching annotated proteins by sequence similarity using BlastP (with a hit e-value cutoff < 1e-20) of the coding protein genes with the GenBank NR, TAIR10 and SwissProt databases (downloaded on 21 July 2014). Additionally, the protein domains were annotated using InterProScan. Functional annotations were integrated using the program AHRD v2.0.2 (https://github.com/groupschoof/AHRD).

The *N. attenuata* assembly and annotation were used as provided by the *N. attenuata* sequencing project [51]. The *N. benthamiana* database was built with the *N. benthamiana* genome version 1.0.1 [52].

### Database build and curation

The setup of PGDBs has been described in more detail previously [30]. In short, the Pathway Tools software component Pathologic was used to initialize new databases and run the builds from specially formatted annotation files that were created using custom Perl scripts. The process is described in detail in the Pathway Tools User’s Guide. Curation has been carried out by extracting information about pathways, reactions, genes, enzymes and compounds from peer-reviewed resources (see more details in the Results and Discussion section), strictly adhering to the guidelines of the curator guide for PGDBs [53]. A validation process of the predicted metabolic network of *Nicotiana* species has been carried out, resulting in adding appropriate new pathways and removing incorrectly predicted pathways. A blacklist of known false-positive pathways generated from previous annotation efforts [30] was applied to *Nicotiana*-specific databases during the confirmation process to correct the predicted metabolic network of the particular species.

### Figures and tables

Figures and tables were created with the 2016 version of Microsoft Word, Excel or PowerPoint and saved in pdf and jpeg format. The Venn diagram was generated using an online tool at the Bioinformatics & Evolutionary Genomics webpage (http://bioinformatics.psb.ugent.be/webtools/Venn/).

## Results and discussion

### The curation process in SolCyc databases

The main curation effort in SolCyc databases is directed toward enriching metabolic pathways with experimentally verified data for genes, enzymes, compounds and reactions. Of the 10 main metabolic categories defined in MetaCyc-derived databases, basic biosynthetic pathways account for close to 60% of the curated data in selected taxon-specific databases. The remainder can be assigned to the other categories, whose contributions are usually in the single-digit percentage range (Supplementary Table S1). These data are consistent with previously reported results [30]. MetaCyc constantly extends the breadth and depth of objects curated in the database [54], which also contains cellular processes such as histone modification and deoxyribonucleic acid (DNA) and ribonucleic acid (RNA) metabolism that have been curated for both bacteria and mammals but not for plants. All changes are routinely propagated to our SolCyc databases, reviewed and implemented where they apply.

The curation process in Solanaceae metabolic databases derived from the MetaCyc reference database follows the recommendations outlined in the curator guide for PGDBs [53]. The SolCyc databases are open source databases and as such open to review by the public and scientific community. Biocuration is an important part of managing the process of biological information with the overall objective to share data of value with scientists in respective research areas. The value of data is generally defined by its correctness, integrity, accessibility, scope and ease of use [55]. As researchers increasingly rely on curated data in the transformation of communication and information to the digital era, we strive to make sure to consistently provide data of high quality and dependability. The curation effort is focused on the goal to provide data pertinent to the scope of the database and to make these repositories valuable resources to researchers.

**Figure 1:**
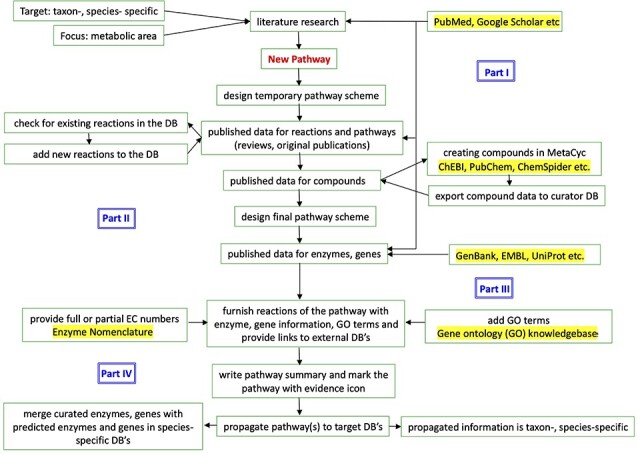
Flow chart of the curation process in the manually curated taxon-specific databases SolanaCyc and NicotianaCyc. External resources used for curation have been highlighted and referenced in the text.

Our main databases for curating pathways are the taxon-specific databases SolanaCyc and NicotianaCyc. In these databases, the creation of new and the revision of existing pathways take place, which subsequently are propagated to the corresponding organism-specific databases for *Nicotiana* species [30]. The curation process for the creation of a new pathway can generally be divided into four parts and is shown in the flow chart of Figure 1. Phase I focuses on extracting information about target species and the desired metabolic area from the scientific literature. External databases such as NCBI’s PubMed (https://www.ncbi.nlm.nih.gov/pubmed) [56] and Google Scholar (https://scholar.google.com/) are valuable resources for finding peer-reviewed original publications and review articles, which allow the retrieval and aggregation of new data that are then used for the design of a temporary pathway layout. In phase II the study of the more specific literature and tapping into external databases provides information about reactions, enzymes, genes and compounds. For the validation of chemical compound structures various resources such as ChEBI [57], ChemSpider [58, 59] or PubChem [60] are used. In order to maintain the high curation standard for the accuracy of chemical structures and exchangeability of data between PGDBs, all compounds are created in MetaCyc and subsequently exported into the SolCyc databases. Before each new MetaCyc release, all compounds are subjected to rigorous structural checks to ensure consistency and correctness of the chemical structures in the database.

For the final draft of the newly created pathway, MetaCyc is checked for existing reactions, which, if needed, are exported into the databases. New reactions are created in the curation databases SolanaCyc and NicotianaCyc, where also the final assembly of the pathway takes place. Published data for enzymes and genes obtained from the literature are cross-checked with external databases, for example GenBank [61] and UniProt [62], and linked to respective reactions. In phase III the newly created pathways are furnished with the extracted chemical, enzymatic and genetic information. Furthermore, gene ontology terms [63] are used to annotate the molecular function, biological process and sub-cellular location of gene products. Enzymatically catalyzed reactions are associated with full or partial EC numbers according to the guidelines of the Enzyme Nomenclature (NC-IUBMB) [64] to characterize the activity of the catalyzing enzyme. In the last phase, phase IV, the pathway is finalized by adding concise comments to the various pathway elements, e.g. enzymes and genes, and the pathway itself. Moreover, links to external resources and publications for curated data are displayed on element-specific detail pages to provide access to the original source. After the propagation of the pathway with the appropriate information to the corresponding *Nicotiana*-specific database, genes and enzymes with sequence information are blasted against the database and merged with the matching predicted counterparts of that database.

### Development of taxon-specific databases at SGN

The SolanaCyc database was the first manually curated database created at SGN using the Pathway Tools suite software package [13, 14] and MetaCyc [15, 16] as metabolic reference database. The inception of the family-level SolanaCyc database took place in December 2015, followed by the creation of the second taxon-specific database NicotianaCyc in June 2016. An overview of the growth of the two databases in-depth and breadth from their initiation (version 1.0) up to the current release version 2.6 in October 2019 is given in Table 1. Due to the higher number of pathways available from MetaCyc for all members of the nightshade family, SolanaCyc started out with a better basic coverage of metabolic data than the genus-specific NicotianaCyc. Between version 1.0 and version 2.6, the number of metabolic pathways in SolanaCyc slightly increased by 97 metabolic pathways, which was somewhat lower than the growth in NicotianCyc with 125 newly added metabolic pathways. Currently, the curation in all our databases focuses on *Nicotiana* species, which explains the overall higher numbers for the increase of metabolic data accrued in NicotianaCyc. A special effort was made in the curation of transporters responsible for the uptake of heavy metals, nutrients and specialized metabolites and their distribution within plants, which is reflected in the high number of curated transporters. Heavy metals are systemic toxicants and classified as known or probable human carcinogens [65]. Heavy metals affect human health through oxidative stress, DNA damage and the acceleration of cell death, which leads to a higher susceptibility to cancer and cancer-like diseases [66]. Plants are known to accumulate heavy metals, which means that these compounds are often ingested through plant-based foods. On the other hand, this ability of the plants to accumulate is used in the phytoremediation of heavy metals from contaminated soils [67]. Both aspects underline the importance of knowing how heavy metals are absorbed and distributed in plants.

**Table 1:** Summary of numbers of pathways, enzymes, protein complexes, transporters and compounds curated in SolanaCyc and NicotianaCyc in the release version 2.6 (2019) in comparison with the first released version 1.0 (2016)

Database		Pathways	Enzymes	Protein complexes	Transporter	Compounds
*SolanaCyc*	1.0	169	257	35	0	1441
	2.6	266	663	75	73	1931
*NicotianaCyc*	1.0	18	32	0	0	260
	2.6	143	273	26	32	848

**Figure 2:**
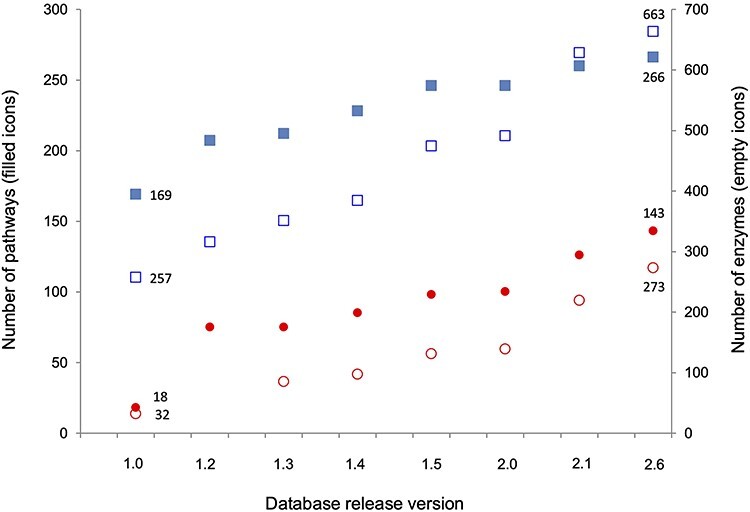
Number of pathways and curated enzymes in the taxon-specific databases SolanaCyc and NicotianaCyc. Square icons represent pathways (filled) and enzymes (empty) of the family-specific SolanaCyc database and round icons represent pathways (filled) and enzymes (empty) in the genus-specific NicotianaCyc database. Note that transporters and enzyme complexes have been excluded from the protein count.

The cumulative statistics for each release, for both curated pathways and enzymes in SolanaCyc and NicotianaCyc, are shown in Figure 2. Currently the 143 pathways curated for Nicotiana species account for 53.8% of all the pathways present in the family-specific database SolanaCyc, which is a significant increase from the 10.7% ascertained in version 1.0. Similarly, the percentage of curated *Nicotiana*-specific enzymes in SolanaCyc increased from 12.4 to 41.2%. In other words, although in version 1.0 about every 8th curated enzyme was contributed by *Nicotiana* species, version 2.6 shows that every 2.4th enzyme now belongs to *Nicotiana*-curated enzymes.

The comparison of curated pathways between 2016 and 2019 (Supplementary Table S2) shows the growing number of curated pathways for the various species contributing metabolic data to SolanaCyc. Not unexpectedly, the highest increase of 91.7% in the number of curated metabolic pathways has been realized for *N. tabacum*, followed by *Solanum lycopersicum* pathways with a rise of 59.8%. Also, the increase of curated pathways associated with a number of economically important species gathered in the SolanaCyc database such as *S. tuberosum* (44.6%), *Petunia x hybrida* (52%) and *Capsicum annuum* (50%) is significant. Among the *Nicotiana* species, 20 new pathways have been added to the species-specific database of *N. benthamiana* and 13 new pathways to the newly created PGDB of *N. attenuata*. Moreover, the coverage of species in the two taxon-specific databases has also been significantly improved. Previously, 27 species were covered, which comprised 9 genera in SolanaCyc, and the 8 species recorded in NicotianaCyc in version 1.0 [30]; these numbers have now risen to 42 species of 12 genera in SolanaCyc and 16 species in NicotianaCyc.

**Figure 3:**
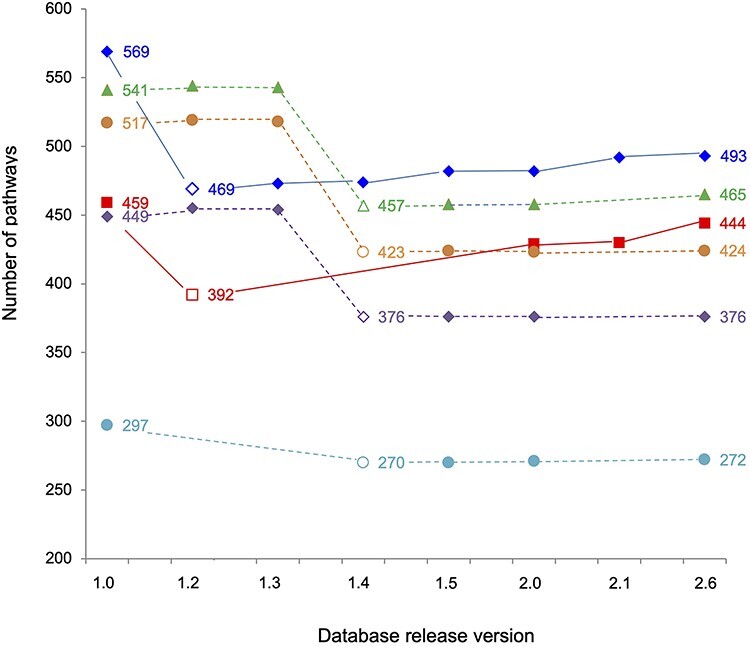
Curation progress in *Nicotiana*-specific databases over time. Filled icons represent the number of pathways present at the time of the respective release version. Empty icons mark the release version of the blacklist application referenced in the text. 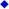
*N. tabacum* (TN90) 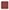
*N. tabacum* (K326) 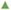
*N. benthamiana*
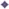
*N. sylvestris*
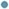
*N. attenuata*
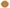
*N. tomentosiformis*.

The impact of manual curation on the species-specific databases of *Nicotiana* is demonstrated in Figure 3. With the exception of the PGDB of *N. tomentosiformis*, a species for which no published pathway could be identified, the remaining 5 *Nicotiana*-specific databases were extensively curated and can, according to the BioCyc definition of curated databases, be considered as a Tier 1 category database [15]. The course of the curation curve for pathways in Figure 3 shows a significant drop of pathway numbers for the two *N. tabacum* databases of TN90 and K326 for the version 1.2 and for the remaining *Nicotiana* databases in version 1.4. This is related to the fact that at those times, a blacklist of metabolic pathways was applied, containing pathways that had been previously found to be invalid for solanaceous species. Although those pathways had been predicted by Pathway Tools using MetaCyc as reference, 156 pathways were invalidated as they have not been reported to occur in *N. tabacum* and other members of the Solanaceae family [30]. MetaCyc is a multi-specific database that curates pathways from all kingdoms of life and, based on the metabolic information collected, predicts metabolic networks in respective PGDBs. Many of the predicted pathways considered invalid for solanaceous plants had been identified through in-depth validation and confirmed to be specific for bacteria, fungi or metazoa, whereas others were pathways for specialized metabolites clearly not occurring in the nightshade family.

The recent curation process for the SolCyc databases (version 2.6) includes updating this blacklist of invalid pathways, supplemented by new pathways from MetaCyc that were erroneously imported during the automated prediction process. Out of 27 new pathways, 7 were declared invalid, 5 are specific to bacteria or Mammalia, one produces a specialized metabolite not present in *Solanaceae* and one lacks a key enzyme that is only found in lower photosynthetic organisms, but not in the highly evolved members of the *Solanaceae* family.

Figure 3 shows a steady increase of pathway numbers for both *N. tabacum* TN90 and K326, as well as for *N. benthamiana*. The progress of adding pathways to the *N. attenuata* database is not so obvious, due to the fact that *N. attenuata* specific pathways had already been curated before the creation of the database and deposited in NicotianaCyc. When the genome of *N. attenuata* became available [51], the corresponding database was created and updated with the curated metabolic pathways stored in NicotianaCyc in advance to the later conducted blacklist check.

### Curation progress: an example—plant cholesterol biosynthesis

In contrast to animals, plants generally contain only diminutive amounts of cholesterol of about 1–2% of total sterols. As a consequence, the pathway was well characterized in animals but poorly understood in plants [68]. However, solanaceous plants can accumulate cholesterol to significantly higher levels [69] and may reach up to 12% of total sterols in *N. benthamiana* [70]. It was not until 2014 that the first committed enzyme involved in the biosynthesis of cholesterol in plants was characterized. This enzyme, the sterol side chain reductase 2 (SSR2) catalyzes a branch point reaction that channels the common precursor cycloartenol toward the cholesterol biosynthetic pathway instead to the formation of C-24 alkylsterols [71]. That was the first indication that cholesterol biosynthesis occurs in plants, which in turn also supported studies that implicated the involvement of cholesterol in the biosynthesis of steroidal glycoalkaloids [72, 73]. At this time, the curation of the cholesterol pathway contained the one confirmed reaction as shown in Figure 4A, together with the conversion of another compound accepted by SSR2, i.e. desmosterol, which, however, is an intermediate for cholesterol in animals and does not occur in plants. Revisiting the pathway, after new literature pertinent to the cholesterol biosynthesis was published, allowed for the full elucidation of this pathway in plants. Eventually, the entire set of cholesterogenesis genes was identified and used to decipher the reaction sequence required for the biosynthesis of cholesterol in plants [74, 75]. This is represented in the updated version of the cholesterol biosynthesis pathway (Figure 4B), which can now be freely accessed in both MetaCyc (https://metacyc.org/META/NEW-IMAGE?type=PATHWAY&object=PWY18C3-1) and SolCyc databases (http://solcyc.solgenomics.net/SOLANA/NEW-IMAGE?type=PATHWAY&object=PWY18C3-1).

**Figure 4a:**
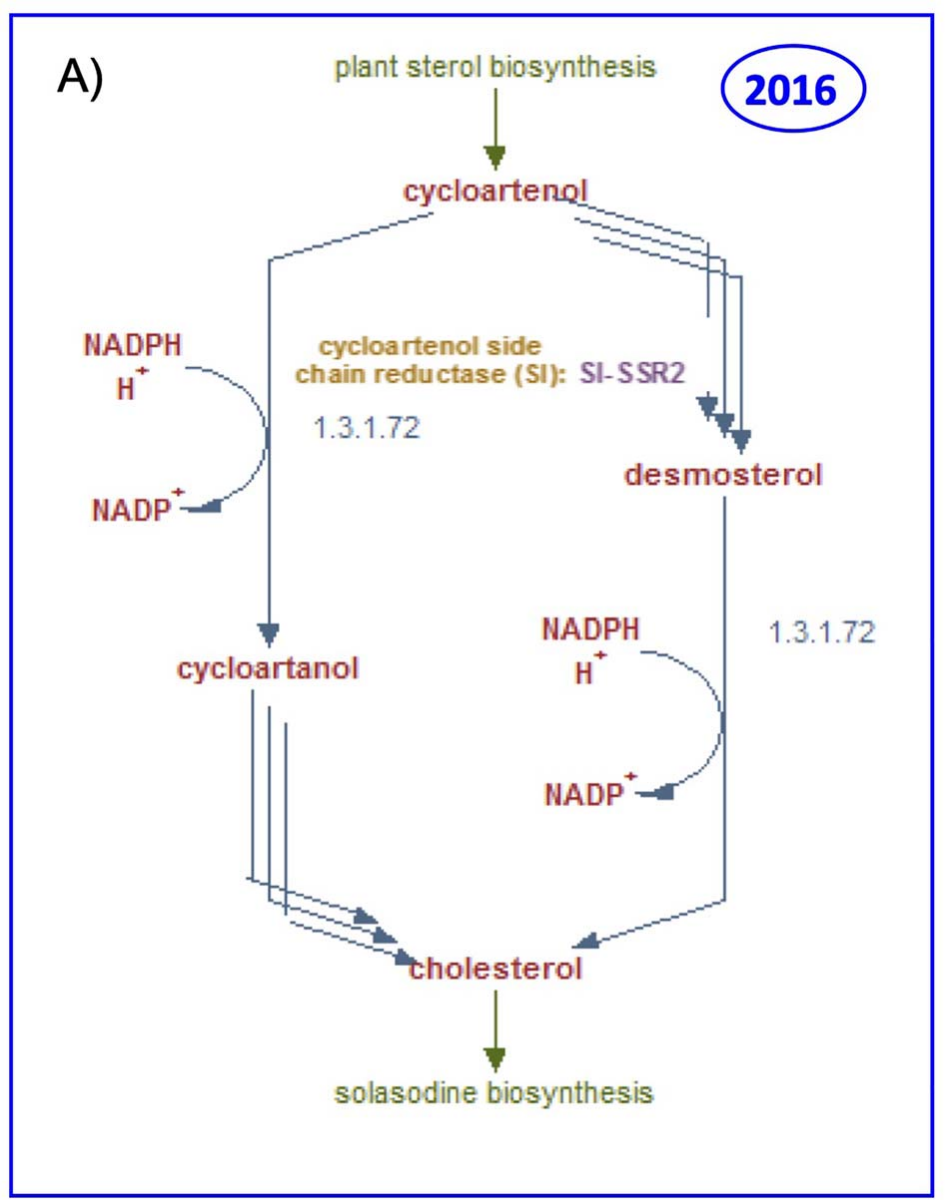
Curation progress made between 2016 (**A**) and 2019 (**B**) on the cholesterol biosynthetic pathway in solanaceous plant species.

**Figure 4b:**
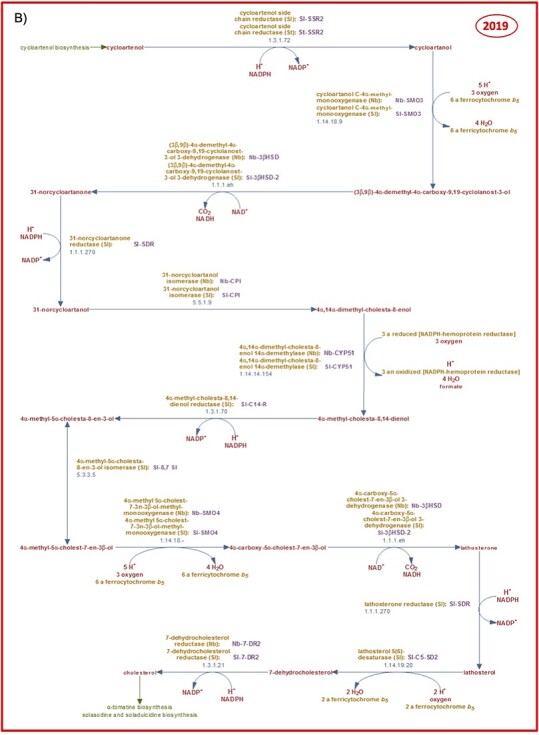


Despite earlier beliefs that cholesterol may not be a plant metabolite, free cholesterol and cholesterol esters have been detected in leaves and organelles of various plants [76], a finding that indicates that cholesterol is more widespread in the plant kingdom than originally thought. The probable occurrence of metabolic pathways in plants is indicated in SolCyc databases by the expected taxonomic range. In accordance with the relevant literature, the distribution of cholesterol biosynthesis was assigned to the taxonomic rank of the *Viridiplantae*. To our knowledge, with the exception of enzymes and coding genes characterized in *S. lycopersicum* and *N. benthamiana*, no reports of enzymes from other plant species involved in the cholesterol biosynthetic pathway have been published. This is not entirely surprising since cholesterol is the starting substrate from which steroidal glycoalkaloid pathways such as the solasodine (http://solcyc.solgenomics.net/SOLANA/NEW-IMAGE?type=PATHWAY&object=PWY18C3-2) and alpha-tomatine (http://solcyc.solgenomics.net/SOLANA/NEW-IMAGE?type=PATHWAY&object=PWY18C3-3) biosynthesis are derived [72, 73] and form compounds that are typically found in members of the *Solanaceae* family. Further research into cholesterol biosynthesis in plants outside the *Solanaceae* family is required to assess the general validity of the metabolic pathway in the plant kingdom or the classification of this metabolic pathway as a family or species-specific variant for the *Solanaceae*.

Many of the identified cholesterol biosynthetic enzymes were shown to behave in a promiscuous way and are equally active in related pathways such as the C-24 alkylsterol biosynthesis. Enzyme promiscuity is often a hallmark of the formation of specialized metabolites. The operation of enzymes catalyzing reactions in both cholesterol and C-24 alkylsterol pathways agrees with the evolutionary ‘patchwork’ hypothesis, which proposes that duplication and diversification events propel the evolution of ancestral enzymes with low substrate specificity into enzymes of new catalytic properties, which leads to the genesis of new pathways [42, 77, 78].

The pathway diagram of cholesterol biosynthesis provides the visualization of reactions, converting precursor compounds in a designated reaction sequence, associated enzymes and genes, and EC numbers and hyperlinks to related pathways. A ‘zoom-in’ functionality allows more detailed pathway information to be displayed and gives access to the detail pages of the various pathway elements. All of the curated enzymes are furnished with comments and links to external databases used for curation. The pathway summary finally provides a concise description of the salient points of the pathway, such as rate-limiting steps, key enzymes and regulation or stereochemistry of involved compounds, and lists the cited literature with links for access to the full publication (Figure 4B).

### Coverage of pathways and enzymes in curated SolCyc databases

The release of MetaCyc (version 22.6) [16] listed 15 263 compounds in the database, which corresponds to about 1.5% of the 1 million metabolites estimated to exist in plants [38]. The arrival of new technologies permitting a much easier interpretation of big data obtained from transcriptomics, metabolomics and genomics experiments [79] opens the door for the discovery of novel reactions and pathways in plants [80]. Despite such new developments, we only possess a very limited knowledge about the underlying biosynthesis, which has been projected to cover about 0.1% of all biochemical pathways [81, 82]. It has been reported that single plant species can synthesize between 5000 and 20 000 primary and specialized metabolites [35]. As a consequence, even the best-curated databases can currently only provide a glance at the entirety of plant metabolism. However, continued development and improvement of such databases by compiling the shared information from the scientific community will generate more comprehensiveness and is considered as an effective tool toward appreciable progress in understanding plant metabolism [83, 84].

The coverage of curated enzymes shown in Table 2 in *Nicotiana*-specific databases also reflects this current status. Among the species-specific databases for *Nicotiana*, proteins, predicted to function as enzymes, accounting for a share of all encoding genes in a range of 17.87% (*N. sylvestris*) to 32.93% (*N. attenuata*). Of those predicted enzymes, the percentage of curated proteins ranges between 0.079% (*N. sylvestris*) and 1.01% (*N. tabacum* TN90). A much higher percentage of curated enzymes versus predicted enzymes can be found in databases for the model organisms *Saccharomyces cerevisiae* (32.04%) and *Homo sapiens* (25.79%). It is important to note that the number of genes in the yeast genome is 5–12 times smaller than in *Nicotiana* species. Apart from being much better studied in the literature than any other organism, the human genome, with about 20 000 genes, is also significantly smaller than any of the *Nicotiana* genomes.

**Table 2:** The degree of pathway and enzyme coverage in curated species-specific *Nicotiana* databases and selected databases of the BioCyc database collection

Database/version	Genes	Predicted enzymes (%)	Curated enzymes (%)	Database host
*N. attenuata*/ 2.6	13 206	4349 (32.93)	12 (0.28)	Sol Genomics Network
*N. benthamiana*/ 2.6	57 142	12 511 (21.89)	23 (0.18)	Sol Genomics Network
*N. sylvestris*/ 2.6	35 536	6349 (17.87)	5 (0.079)	Sol Genomics Network
*N. tabacum* TN90/ 2.6	69 349	19 655 (28.34)	199 (1.01)	Sol Genomics Network
*N. tabacum* K326/ 2.6	69 401	20 227 (29.15)	199 (0.98)	Sol Genomics Network
*N. tomentosiformis*/ 2.6	34 379	9257 (26.92)	0 (0)	Sol Genomics Network
*S. cerevisiae*/ 22.6	5846	1286 (22.75)	412 (32.04)	BioCyc Database Collection
*H. sapiens*/ 22.6	20 578	3652 (17.74)	942 (25.79)	BioCyc Database Collection

A similar picture as seen for the *Nicotiana* database curation emerges when comparing the coverage of curated enzymes versus predicted proteins of other plant species (Supplementary Table S3). Except for *Arabidopsis thaliana*, which is by far the best-curated plant species in the plant kingdom, and which accounts for 25.65% of curated proteins in their database, all other species from intensively curated plant databases such as rice, tomato, potato and corn are essentially in the same percentage range as the *Nicotiana*-specific databases. All of the listed databases in Supplementary Table S3 have been built with Pathway Tools and MetaCyc as reference database. The Gramene database was developed using the data structure of the Human Reactome project [5] and *Oryza sativa* as reference species. The curated genetic information from this database was used to derive orthology-based projections for other species, including crops and model plant species [85]. Although the Gramene database is structured differently to BioCyc databases and concentrates primarily on collecting information on individual reactions and their catalyzing enzymes, it provides valuable metabolic, genetic and genomic information [86, 87]. Such data enrich their own collection of plant metabolic pathway Cyc’s hosted at Gramene (http://pathway.gramene.org/) and complement the efforts of other metabolically oriented databases to get a more complete picture of plant metabolism.

The value of a database depends very much on the quality and reliability of the stored data, which in turn indicate the usefulness of a database equipped with bioinformatics tools that allow querying, visualizing and analyzing tasks [88, 89]. One measure of high-quality data is the extent to which literature, based on experimental results, has been included in the curation process [90]. Table 3 summarizes to what degree pathways are supported by literature across a number of databases. High average numbers of processed literature per pathway have been found in MetaCyc (19.12), SolanaCyc (18.24) and NicotianaCyc (22.21), which corresponds to the high curation levels (99.9–100%) in those databases. Somewhat lower numbers of 9.37 publications per pathway have been determined in PlantCyc, which also reflects a high standard of curation (95.9%). Databases with a lower curation status such as *S. tuberosum*, *S. lycopersicum* and most of the *Nicotiana*-specific databases range an average of about 3–4 publications per pathway. The *N. tabacum* databases for the TN90 and K326 cultivar score a higher average number of 5–7 publications per pathway and also show a higher number of curated pathways of about 25% in comparison with at most 5% curated pathways in the remaining *Nicotiana* databases. The percentage of curated pathways refers to the number of paths with experimental evidence for which existence in the metabolic network of this organism is proven. Pathways predicted and propagated from MetaCyc to species-specific databases such as the *N. tabacum* databases are transferred without enzymes, genes or evidence codes curated in MetaCyc. After validating such pathways, findings from the published literature are used to furnish the pathways with the genes and enzymes of the respective species, which only then justifies marking the pathway as experimentally verified.

**Table 3:** Number of publications used for curating pathways in manually curated metabolic databases covering either multi-species (MetaCyc, PlantCyc, SolanaCyc, NicotianaCyc) or single-species (Nicotiana- and Solanum-specific Cycs) PGDBs

Database (version)	Base pathways	Superpathways	Citations	Average number of citations per pathway	Percentage (%) of curated pathways	Number of species
MetaCyc (22.6)	2698	384	58 954	19.12	99.9	2980
PlantCyc (13.0)	1013	110	10 518	9.37	95.9	366^*^
SolanaCyc (2.6)	266	36	5511	18.24	100.0	42
NicotianaCyc (2.6)	143	21	3642	22.21	100.0	16
*N. attenuata* (2.6)	272	31	1822	6.01	5.2	1
*N. benthamiana* (2.6)	465	61	1891	3.60	4.5	1
*N. sylvestris* (2.6)	376	50	1616	3.79	1.3	1
*N. tabacum* TN90 (2.6)	493	64	3368	6.05	25.6	1
*N. tabacum* K326 (2.6)	444	48	3366	6.84	28.4	1
*N. tomentosiformis* (2.6)	424	59	1587	3.29	0	1
*S. lycopersicum* (3.3.2.2)	455	62	1604	3.10	0^#^	1
*S. tuberosum* (2.2.2)	420	57	1671	3.50	0^#^	1

The number of species in PlantCyc (^*^) has been calculated from release version 11.0. The percentages of curated pathways marked with (^#^) belong to databases which have not yet been updated with curated data stored in SolanaCyc.

The results summarized in Table 3 must consider two factors. First is the observation that the respective databases cover different taxonomic areas. Although MetaCyc includes curated metabolic data from 2980 species across all kingdoms and PlantCyc covers the same for more than 350 plant species, SolanaCyc and NicotianaCyc contain the metabolic content from 42 and 16 species, respectively. It is therefore not unexpected that species-specific databases contain far fewer curated pathways due to the high number of predicted pathways with only marginal literature support. The high percentage of curated pathways in the multi-species MetaCyc reflects the fact that the pathways for one or more of the species in the database have been experimentally verified. This determination still has to be made in species-specific databases, which leads to a lower percentage of curated pathways due to highly specific and therefore more limited information available for this particular species. The second consideration is the period during which these databases were curated. MetaCyc has been in existence for 20 years, whereas PlantCyc was established in 2008. The curation of the first *Nicotiana* specific database started 2016. It can therefore be concluded that in the comparably short time, in which the databases of SolCyc have been curated, the approach of utilizing taxon-specific databases such as SolanaCyc and NicotianaCyc for the metabolic enrichment of *Solanum* and *Nicotiana* specific databases has been successful.

### Data downloads and portability

Downloading, transportability and programmatic access to data in databases created with Pathway Tools are provided via Application Programming Interfaces (APIs), including web-based analysis tools. Many of those tools are available in desktop and/or web server modes and Pathway Tools maintains a webpage of those services (https://biocyc.org/web-services.shtml). Downloads of BioCyc databases of interest are available but require the purchase of a license (https://biocyc.org/download.shtml). Programmatic interfaces for the Pathway Tools software at the SGN can be found here (https://solgenomics.net/downloads/index.pl). Data in all databases created by Pathway Tools, such as the SolCyc databases, can be accessed via a variety of search and analysis options. A comprehensive description of these tools has been published [13] and is updated regularly [14]. Many of the data obtained by search and analysis can be stored in a SmartTable [22] and accessed in JSON, XML or tab-delimited format. Pathway graphs or the metabolic overview with highlighted data can be exported to the Pathway collage tool [21] and saved in PNG format. Pathways accessed via web service can also be saved in BioPAX format for further analysis and display. Some of those tools and applications in Nicotiana-specific databases have been discussed previously [91].

### Current status of metabolic networks at SGN

The complexity of metabolic networks originates in the many thousands of enzymes, specifically functioning within the confines of the metabolic range that exists in individual plant species in a time and space dependent manner. A quantitative analysis of the proteome of pollen from tomato alone resulted in more than 1200 proteins [92], whereas 500–600 proteins were detected in the various ripening stages for tomato fruits and more than 1000 proteins in leaves [93]. Protein knowledge databases such as UniProt [62] lend huge support to curators of all biological databases. Plant species, which receive the highest amount of curation in SolCyc databases, such as *N. tabacum* and *S. lycopersicum* are well represented in UniProt and were among the six top-ranked plant species with the most protein entries at the time of this study [94]. UniProt had been reported to contain more than 60 million unique protein sequences in 2016 [95]. At present, the database (https://www.uniprot.org/) hosts more than 147 million protein sequences, among them close to 77 000 unreviewed protein data and 855 entries for reviewed proteins of *N. tabacum*. This resource of protein knowledge is invaluable for the curation in databases like the SolCyc collection of databases. Not only is UniProt a repository for the search of relevant enzymes published in the literature, but it also aids the process of authentication and matching of confirmed protein sequences with the predicted protein sequences embedded in the metabolic network of genome annotated species.

**Figure 5:**
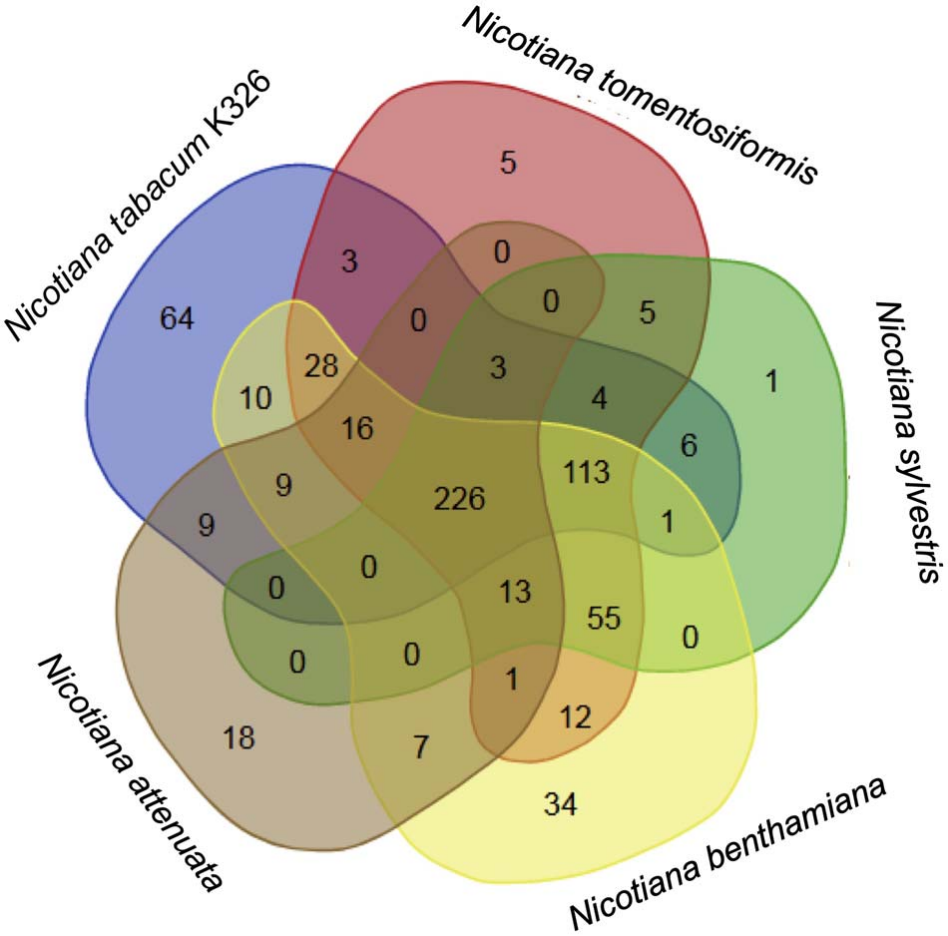
Nicotiana-specific pathway network Venn diagram. Crossover of pathway networks between *N. tabacum* (K326), *N. sylvestris*, *N. tomentosiformis*, *N. benthamiana* and *N. attenuata*. The numbers for shared and unique pathways are shown in each intersection.

The metabolic composition of networks of *Nicotiana* species has already been reported to be in good agreement with each other [30]. However, the comparison of the pathways contained in each individual metabolic network (Figure 5) also accentuates the differences between the various *Nicotiana* species. The Venn diagram (http://bioinformatics.psb.ugent.be/webtools/Venn/) for the entirety of pathways in *N. tabacum* K326, *N. attenuata*, *N. benthamiana*, *N. sylvestris* and *N. tomentosiformis* highlights areas of shared metabolism and a number of pathways that are specific for each *Nicotiana* species. The intersection for the metabolic pathways of *N. tabacum* K326 and its parental species *N. tomentosiformis and N. sylvestris* is 59.3%, which further drops with the addition of *N. benthamiana* and *N. attenuata* pathways to 35.1%. The comparison of *N. tabacum* K326 pathways with the metabolic networks of *N. benthamiana* and *N. attenuata* shows an overlap that is about 20% lower than the one observed with the parents of *N. tabacum*. That indicates the greater evolutionary distance of those species to *N. tabacum* K326. The number of unique pathways not shared with any of the studied species is highest in *N. tabacum* (64), followed by *N. benthamiana* (34), *N. attenuata* (18), *N. tomentosiformis* (5) and *N. sylvestris* (1). A closer look at the nature of the pathways (results not shown) revealed that the majority of those pathways (34) belong to specialized metabolites. To a large extent, this also includes newly created pathways (23), which probably are valid for other *Nicotiana* species as well, but for which only enzymes from *N. tabacum* have been published. The existing gap in shared pathways will get smaller when metabolic information from the remaining *Nicotiana* species become available. However, a certain amount of divergence for some metabolic categories in domesticated and wild tobacco species is to be expected. The genus *Nicotiana* is known for its high molecular diversity due to the polymorphic variability of its species, often resulting in either gaining or losing biosynthetic flexibility [96].

### Conclusions and prospect for curated taxon-specific databases

Databases of all types have been widely accepted as efficient tools to aid researchers with bioinformatics analyses and visualization of stored and annotated data. The value of data directly depends on their overall quality, best achieved by the ‘gold standard’ manual curation by trained biocurators with the ability to distill the published literature to meaningful data through critical assessment [97]. Biocurators are also the professionals that transform data into database-compliant information that can be easily extracted and used with the database’s various bioinformatics tools [4]. For instance, the improvement in accuracy of prediction models for drug discovery trained with manual curated data [98] or the curation of genetic variants employing curated biomedical literature [99] as well as the detection of misannotated enzymes through curation [100] has proven the value of manually curated data. However, manual curation of databases is not undisputed. In comparison with the immense increase in the amount of data published in journals or online, the proportion of curated data is getting smaller and smaller, urging proposals for the advancement of automated text mining [101]. Nevertheless, studies showed that manually curated databases are much less prone to contain errors than databases employing automated or semi-automated text mining tools [97]. For instance, the *Escherichia coli* (EcoCyc) database and the Candida Genome Database, both manually curated, had individual error rates of 1.4 and 1.8%, respectively, [102] compared with the 14–42 times higher error rates in databases utilizing information-extraction programs [97]. When too many errors were found through the automated prediction of metabolic pathways for the functional genomic database of the Malaria parasite *Plasmodium falciparum*, manual validation was essential to accept or reject predicted pathways for correctly reconstructing the metabolic network of this species [103]. Manual curation performed by biocurators was critical to the accuracy and completeness of the Saccharomyces genome database [104]. *Saccharomyces cerevisiae* was also the subject of a study that manually curated the growth and cell cycle interaction network and provided detailed insights into the relationships between these developmental processes [105].

It has been projected that manual curation cannot achieve functional gene annotation in humans or keep up with the rate of newly sequenced genomes [106]. Such concerns have been raised previously, but at the same time it has been stressed that around 30% of the human genome, which cannot be correctly predicted by automated approaches, requires manual curation and will be invaluable to researchers who work with human metabolism [107]. Indeed, selected aspects of metabolism have been successfully accomplished by manually curation in humans such as targeted curation of a molecular interaction database [108], the curation of human fatty acid metabolism [109] and the manually curation of RNA-binding protein involved in human RNA metabolism [110]. Another example highlighting the value of manual curation is the identification of proteins and corresponding metabolic pathways that caused lead poisoning in humans [111].

New approaches need to be taken to solve the lack of trained biocurators and funding of manual curated databases versus the tremendous data input coming from the literature [112, 113]. On the other hand, authors publishing experimental data that can support the pathways information should be required to provide the metadata in formats that can be automatically read into pathway databases such as MetaCyc. This would help biocurators import much larger sets of experimentally verified data through automatic pipelines so they can focus on quality control.

With the creation of manually curated taxon-specific databases and following an expert-led curation process, databases assembled under the umbrella of SolCyc have been significantly enriched with high-value data. The curation progress in SolanaCyc and NicotianaCyc has improved the completeness and accuracy of the metabolic networks in the six *Nicotiana*-specific databases. Moreover, it has advanced SolanaCyc and NicotianaCyc to more comprehensive repositories for the biochemistry and molecular biology of the *Solanaceae* family and the *Nicotiana* genus. The data in those taxon-specific databases have been curated with a focus on narrower taxonomic ranges and are therefore more useful for exploring solanaceous metabolic networks and relevant bioinformatics analyses. These achievements can now be leveraged into new approaches, such as multi-species databases containing not only plant host but also their pathogens to more precisely and effectively model the interactions between host and pathogen.

The new emerging field of pan-genome research, first proposed in the genome analysis of pathogenic isolates of *Streptococcus agalactiae* [114], opens the possibility to study the genetics of selected species or a phylogenetic clade in a comparative way [115, 116]. We plan to expand and transfer the concept of pan-genomics to pan/meta-metabolomics and create a database that contains the metabolic networks of *Nicotiana* species and disease-causing microorganisms. We expect this MetaTobaccoCyc database to be helpful in exploring the metabolic diversity and commonality between *Nicotiana* and interacting species by defining metabolic subsets of core and specific pathways and their catalyzing enzymes, which may be indicative for adaptive evolutionary developments in individual metabolomes of *Nicotiana* species and known pathogens for the genus.

**Key Points**

- Metabolic databases transform, evaluate and visualize Omics- and high-throughput data into the biological context of species and allow bioinformatics analyses in diverse areas of interest.
- Manually curated metabolic databases increase the accuracy and comprehensiveness of data and substantially lower the rate of misannotations and false-negative results in predicted metabolic networks.
- SolanaCyc and NicotianaCyc are the first manually curated taxon-specific databases for the *Solanaceae* family and *Nicotiana* genus, respectively.
- Taxon-specific metabolic databases are valuable and specific repositories for the biochemistry and molecular biology of defined taxonomic ranks.

## Supplementary Material

table_1S_bbaa136

tab_2_bbaa136

Tab_S3_bbaa136

legend_to_figures_and_tables_bbaa136

## Notes

**Hartmut Foerster** is a Research Associate at the Boyce Thompson Institute, where he curates metabolic networks of the Solanaceae.

**James N.D. Battey** is a Computational Scientist at PMI R&D, Philip Morris Products S.A., where he works analyzing next generation sequencing data from genomics, transcriptomics and metagenomics experiments.

**Nicolas Sierro** is the Manager of the Genomics and Transcriptomics Group at PMI R&D, Philip Morris Products S.A., where he works on the production and analysis of next- and third-generation sequencing data from plant, nonclinical and clinical projects.

**Nikolai V. Ivanov** is a Global Head of Biomarkers and Biosensors at PMI R&D, Philip Morris Products S.A., where he leads the OMICS laboratories producing and analyzing data for plant, clinical and nonclinical projects.

**Lukas A. Mueller** is a Professor at the Boyce Thompson Institute, where he studies plant genomes and breeding databases.
