# Supplementary material for: Metabolic networks of the Nicotiana genus in the spotlight: content, progress and outlook
Source: Brief Bioinform. 2020 Jul 14;22(3):bbaa136. doi: 10.1093/bib/bbaa136 (PMC8138835; doi:10.1093/bib/bbaa136)
Supplement: table_1S_bbaa136 [file table_1s_bbaa136.docx]

|  | **SolanaCyc** (2.6) | | **NicotianaCyc** (2.6) | | **TobaccoCyc** (2.6) | |
| --- | --- | --- | --- | --- | --- | --- |
|  | # of pwys | % | # of pwys | % | # of pwys | % |
| **activation/ inactivation/ interconversion** | 7 | 2.32 | 5 | 3.05 | 10 | 2.03 |
| **biosynthesis** | 178 | 58.94 | 95 | 57.93 | 283 | 57.52 |
| **degradation/ utilization/ assimilation** | 27 | 8.94 | 11 | 6.71 | 70 | 14.22 |
| **detoxification** | 4 | 1.32 | 3 | 1.82 | 9 | 1.83 |
| **precursor metabolites and energy** | 11 | 3.64 | 5 | 3.05 | 20 | 4.06 |
| **glycan pathways** | 4 | 1.33 | 3 | 1.83 | 14 | 2.85 |
| **macromolecular modification** | - | - | - | - | 3 | 0.61 |
| **metabolic clusters** | 16 | 5.30 | 6 | 3.66 | 21 | 4.27 |
| **transport** | 19* | 6.29 | 15** | 9.15 | 14 | 2.85 |
| **superpathways** | 36 | 11.92 | 21 | 12.80 | 48 | 9.76 |

**Table S1**. Assignment of pathways to the metabolic categories of metabolic networks of the family-specific SolanaCyc, the genus-specific NicotianaCyc, and the species-specific TobaccoCyc (Nicotiana tabacum K326) databases. The number of transport pathways marked with (*) includes four pathways curated for non-Nicotiana species, and (**) refers to the number of Nicotiana transport pathways that include one pathway specifically curated for Nicotiana benthamiana.
