# Supplementary material for: Metabolic networks of the Nicotiana genus in the spotlight: content, progress and outlook
Source: Brief Bioinform. 2020 Jul 14;22(3):bbaa136. doi: 10.1093/bib/bbaa136 (PMC8138835; doi:10.1093/bib/bbaa136)
Supplement: tab_2_bbaa136 [file tab_2_bbaa136.docx]

| Genus/Species | # of pathways  2016 | # of pathways  2019 |
| --- | --- | --- |
| *Solanum* |  |  |
| *Solanum lycopersicum* | 87 | 139 |
| *Solanum tuberosum* | 56 | 81 |
| *Solanum habrochaites* | 12 | 13 |
| *Solanum pennellii* | 8 | 8 |
| *Solanum aculeatissimum* | 2 | 2 |
| *Solanum melongena* | 2 | 5 |
| ***Solanum nigrum*** | - | 1 |
| ***Lycopersicon hirsutum glabratum*** | - | 3 |
| ***Solanum pinnatisectum*** | - | 2 |
| *Nicotiana* |  |  |
| *Nicotiana tabacum* | 72 | 138 |
| *Nicotiana sylvestris* | 5 | 10 |
| *Nicotiana benthamiana* | 2 | 22 |
| ***Nicotiana attenuata*** | - | 13 |
| ***Nicotiana langsdorffii x Nicotiana sanderae*** | - | 1 |
| ***Nicotiana plumbaginifolia*** | - | 13 |
| ***Nicotiana rustica*** | - | 2 |
| ***Nicotiana suaveolens*** | - | 3 |
| **Nicotiana glutinosa** | - | 2 |
| **Nicotiana noctiflora** | - | 1 |
| **Nicotiana alata** | - | 1 |
| **Nicotiana langsdorffii** | - | 1 |
| **Nicotiana mutabilis** | - | 1 |
| **Nicotiana longiflora** | - | 1 |
| **Nicotiana forgetiana** | - | 1 |
| **Nicotiana bonariensis** | - | 1 |
| *Cestrum* |  |  |
| *Cestrum elegans* | 1 | 1 |
| *Petunia* |  |  |
| *Petunia x hybrida* | 25 | 38 |
| ***Petunia axillaris*** | - | 6 |
| *Capsicum* |  |  |
| *Capsicum annuum* | 14 | 21 |
| *Capsicum chinense* | 2 | 4 |
| *Capsicum frutescens* | 1 | 3 |
| *Capsicum baccatum* | 1 | 1 |
| *Anisodus* |  |  |
| *Anisodus acutangulus* | 2 | 2 |
| *Atropa* |  |  |
| *Atropa belladonna* | 5 | 5 |
| *Hyoscyamus* |  |  |
| *Hyoscyamus albus* | 3 | 3 |
| *Hyoscyamus muticus* | 1 | 1 |
| *Hyoscyamus niger* | 4 | 4 |
| *Datura* |  |  |
| *Datura stramonium* | 6 | 6 |
| *Datura inoxia* | 1 | 1 |
| ***Withania*** |  |  |
| ***Withania somnifera*** | - | 2 |
| ***Brunfelsia*** |  |  |
| ***Brunfelsia brasiliensis*** | - | 1 |
| ***Iochroma*** |  |  |
| ***Iochroma calycinum*** | - | 2 |

**Table S2**. List of curated pathways of solanaceous species present in SolanaCyc. The progress of curation can be seen in the comparison of the respective status of 2016 with 2019. Note that pathways can be associated with more than one species. Species and genera in bold have been added after 2016.
