# Supplementary material for: Metabolic networks of the Nicotiana genus in the spotlight: content, progress and outlook
Source: Brief Bioinform. 2020 Jul 14;22(3):bbaa136. doi: 10.1093/bib/bbaa136 (PMC8138835; doi:10.1093/bib/bbaa136)
Supplement: Tab_S3_bbaa136 [file tab_s3_bbaa136.docx]

| species/version | predicted enzymes | curated proteins | percentage (%) of coverage | database host |
| --- | --- | --- | --- | --- |
|  |  |  |  |  |
| *Arabidopsis thaliana*/ 16.0 | 5255 | 1348 | 25.65 | Araport and PlantCyc |
|  |  |  |  |  |
| *Brassica napus*/ 2.0 | 17646 | 9 | 0.05 | Plant Metabolic Network |
|  |  |  |  |  |
| *Capsicum annuum*/ 1.0 | 7110 | 7 | 0.10 | Plant Metabolic Network |
|  |  |  |  |  |
| *Capsicum annuum*/ 3.3 | 5678 | 15* | 0.26 | Sol Genomics Network |
|  |  |  |  |  |
| *Glycine max*/ 9.0 | 10643 | 70 | 0.66 | Plant Metabolic Network |
|  |  |  |  |  |
| *Oryza sativa*/ 6.0 | 6320 | 73 | 1,16 | Gramene and PlantCyc |
|  |  |  |  |  |
| *Solanum lycopersicum*/ 4.0 | 9048 | 51 | 0.56 | Gramene and PlantCyc |
|  |  |  |  |  |
| *Solanum lycopersicum*/ 3.3.2.2 | 8033 | 193* | 2.40 | Sol Genomics Network |
|  |  |  |  |  |
| *Solanum tuberosum*/ 5.0 | 6365 | 32 | 0.50 | Plant Metabolic Network |
|  |  |  |  |  |
| *Solanum tuberosum*/ 2.2.2 | 5343 | 87* | 1.63 | Sol Genomics Network |
|  |  |  |  |  |
| *Sorghum bicolor*/ 6.0 | 5952 | 14 | 0.24 | Plant Metabolic Network |
|  |  |  |  |  |
| *Triticum aestivum*/ 2.0 | 22437 | 24 | 0.11 | Plant Metabolic Network |
|  |  |  |  |  |
| *Zea mays*/ 16.0 | 7381 | 107 | 1.45 | Gramene and PlantCyc |

**Table S3**. Comparison of predicted versus curated enzymes in the metabolic networks of selected species-specific plant databases. The numbers for curated proteins marked with an asterisk are for proteins that reside in the family-specific SolanaCyc database, but have not yet been propagated to the corresponding species-specific DB’s.
