# Supplementary material for: Metabolic networks of the Nicotiana genus in the spotlight: content, progress and outlook
Source: Brief Bioinform. 2020 Jul 14;22(3):bbaa136. doi: 10.1093/bib/bbaa136 (PMC8138835; doi:10.1093/bib/bbaa136)
Supplement: legend_to_figures_and_tables_bbaa136 [file legend_to_figures_and_tables_bbaa136.docx]

Legend for figures and tables

Figures

**Fig. 1**. Flow chart of the curation process in the manually curated taxon-specific databases SolanaCyc and NicotianaCyc. External resources used for curation have been highlighted and referenced in the text.

**Fig. 2.** Number of pathways and curated enzymes in the taxon-specific databases SolanaCyc and NicotianaCyc. Square icons represent pathways (filled) and enzymes (empty) of the family-specific SolanaCyc database and round icons represent pathways (filled) and enzymes (empty) in the genus-specific NicotianaCyc database. Note that transporters and enzyme complexes have been excluded from the protein count.

**Fig. 3**. Curation progress in *Nicotiana*-specific databases over time. Filled icons represent the number of pathways present at the time of the respective release version. Empty icons mark the release version of the blacklist application referenced in the text.
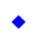
 *Nicotiana tabacum* (TN90)
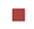
 *Nicotiana tabacum* (K326)
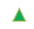
*Nicotiana benthamiana*
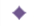
*Nicotiana sylvestris*
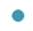
 *Nicotiana attenuata*
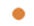
*Nicotiana tomentosiformis*

**Fig. 4**. Curation progress made between 2016 (A) and 2019 (B) on the cholesterol biosynthetic pathway in solanaceous plant species.

**Fig. 5**. *Nicotiana*-specific pathway network VENN diagram. Cross-over of pathway networks between *Nicotiana tabacum* (K326), *Nicotiana sylvestris*, *Nicotiana tomentosiformis*, *Nicotiana benthamiana* and *Nicotiana attenuata*. The numbers for shared and unique pathways are shown in each intersection.

Tables

**Table 1**. Summary of numbers of pathways, enzymes, protein complexes, transporters and compounds curated in SolanaCyc and NicotianaCyc in the release version 2.6 (2019) in comparison to the first released version 1.0 (2016)

**Table 2**. The degree of pathway and enzyme coverage in curated species-specific *Nicotiana* databases and selected databases of the BioCyc database collection.

**Table 3**. Number of publications used for curating pathways in manually curated metabolic databases covering either multi-species (MetaCyc, PlantCyc, SolanaCyc, NicotianaCyc) or single-species (Nicotiana- and Solanum-specific Cyc’s) Pathway/Genome Databases (PGDB’s). The number of species in PlantCyc (*) has been calculated from release version 11.0. The percentages of curated pathways marked with (^#^) belong to databases which have not yet been updated with curated data stored in SolanaCyc.

Supplemental

**Table S1**. Assignment of pathways to the metabolic categories of metabolic networks of the family-specific SolanaCyc, the genus-specific NicotianaCyc, and the species-specific TobaccoCyc (Nicotiana tabacum K326) databases. The number of transport pathways marked with (*) includes four pathways curated for non-Nicotiana species, and (**) refers to the number of Nicotiana transport pathways that include one pathway specifically curated for Nicotiana benthamiana.

**Table S2**. List of curated pathways of solanaceous species present in SolanaCyc. The progress of curation can be seen in the comparison of the respective status of 2016 with 2019. Note that pathways can be associated with more than one species. Species and genera in bold have been added after 2016.

**Table S3**. Comparison of predicted versus curated enzymes in the metabolic networks of selected species-specific plant databases. The numbers for curated proteins marked with an asterisk are for proteins that reside in the family-specific SolanaCyc database, but have not yet been propagated to the corresponding species-specific DB’s.
